# Complete Genome Sequence of *Ralstonia solanacearum* FJAT-91, a High-Virulence Pathogen of Tomato Wilt

**DOI:** 10.1128/genomeA.00900-17

**Published:** 2017-09-14

**Authors:** Deju Chen, Bo Liu, Yujing Zhu, Haifeng Zhang, Zheng Chen, Xuefang Zheng, Rongfeng Xiao, Yanping Chen

**Affiliations:** Agricultural Bio-Resources Research Institute, Fujian Academy of Agricultural Sciences, Fuzhou, Fujian, People’s Republic of China

## Abstract

*Ralstonia solanacearum* FJAT-91, which displays higher virulence toward plants belonging to the family *Solanaceae*, was isolated from a wilted tomato plant vessel in Fujian province, southeast China. Here, we report the complete genome sequence of *R. solanacearum* FJAT-91 using long-read single-molecule PacBio sequencing technology. The genome comprises a 3,873,214-bp circular chromosome and a 2,000,873-bp circular megaplasmid with an overall G+C content of 66.85%.

## GENOME ANNOUNCEMENT

*Ralstonia solanacearum* is a soilborne plant-pathogenic betaproteobacterium with a wide host range that can attack more than 200 plant species belonging to over 50 different botanical families, such as tomato and tobacco, causing plant bacterial wilt (1–3). Direct yield losses by *R. solanacearum* vary widely according to the host, cultivar, climate, soil type, cropping pattern, and strain, (e.g., from 0 to 91% in tomato, 33 to 90% in potato, 10 to 30% in tobacco, 80 to 100% in banana, and up to 20% in groundnut) (4). The extensive genetic diversity of *R. solanacearum* species is responsible for the various plant bacterial wilt diseases. Genome sequence analysis provides clues about the evolution of essential virulence genes, such as those encoding the type III secretion system and related pathogenicity effectors, and it also provides insights into possible mechanisms contributing to the rapid adaptation of the bacterium to its environment in general and to its interaction with plants in particular (5). *R. solanacearum* FJAT-91 was isolated from a wilting tomato vessel in Fujian province, southeast China, and showed high toxicity to plants belonging to the family *Solanaceae*. To further understand molecular mechanisms and explore new strategies for controlling bacterial wilt, we present the complete genome sequence of *R. solanacearum* FJAT-91 using long-read single-molecule PacBio sequencing technology.

FJAT-91 was grown in SPA medium (sucrose, peptone, and agar, pH 7.2–7.4), and genomic DNA was extracted and purified following the manufacturer’s protocols (Pacific Biosciences, Menlo Park, CA, USA). A 20-kb SMRTbell library was prepared from 10 µg of genomic DNA using a 20-kb template library preparation workflow. Single-molecule real-time (SMRT) sequencing was conducted on a PacBio RS II sequencing platform using the C4 sequencing chemistry and P6 polymerase with one SMRT cell. The sequence reaction generated a total of 102,143 subreads and 1,064,333,615 total subread bases, which corresponds to a sequencing coverage of approximately 181×. Sequencing reads were *de novo* assembled following the Hierarchical Genome Assembly Process (HGAP) workflow (PacBioDevNet; Pacific Biosciences) as available in SMRT Analysis version 2.3.1.

Identification of coding sequence and annotation were performed with the NCBI Prokaryotic Genome Annotation Pipeline (see http://www.ncbi.nlm.nih.gov/genomes/static/Pipeline.html), and the complete genome sequence was submitted to the International Nucleotide Sequence Database Collaboration (GenBank).

The complete genome sequence of *R. solanacearum* FJAT-91 comprises a 3,873,214-bp circular chromosome and a 2,000,873-bp megaplasmid, with G+C contents of 66.76% and 67.03%, respectively. The genome contains 4,870 protein-coding genes, 57 tRNAs, 12 rRNAs, and 4 other noncoding RNAs (ncRNAs). All key genes required for the pathogenicity and housekeeping gene are encoded in the FJAT-91 genome. The genome encoded five secretion systems, namely, the type I, II, III, IV, and VI secretion systems. A total of 193 genes were related to the type III secretion system. Furthermore, 326 genes encoded the secreted proteins, including 74 type III effectors, and 86 genes encoded carbohydrate-active enzymes that are related to the degradation of cell walls and other large carbohydrates. The complete genome of *R. solanacearum* FJAT-91 contained a large repertoire of type III effector proteins, which provide material for uncovering the molecular mechanisms of plant wilt, as well as avirulent type III effectors (6, 7). We can introduce avirulent type III effectors into avirulent *R. solanacearum* FJAT-1458 to construct a plant vaccine to facilitate the biocontrol of bacterial wilt in agriculture (8).

### Accession number(s).

The genome information for the chromosome and megaplasmid of *R. solanacearum* FJAT-91 has been deposited in GenBank under the accession numbers CP016612 and CP016613, respectively. The strain can be obtained from the microbiology lab at the Agricultural Bio-Resources Research Institute, Fujian Academy of Agricultural Sciences.
